# Protein stability regulators screening assay (Pro-SRSA): protein degradation meets the CRISPR–Cas9 library

**DOI:** 10.1186/s40880-016-0125-z

**Published:** 2016-06-29

**Authors:** Yuanzhong Wu, Tiebang Kang

**Affiliations:** Sun Yat-sen University Cancer Center, State Key Laboratory of Oncology in South China, Collaborative Innovation Center for Cancer Medicine, 651 Dongfeng Road East, Guangzhou, 510060 Guangdong P. R. China

**Keywords:** CRISPR–Cas9 screening, Protein stability, Cdc25A, Ubiquitination, Acetylation

## Abstract

The regulation of protein stability is a fundamental issue for biophysical processes, but there has not previously been a convenient and unbiased method of identifying regulators of protein stability. However, as reported in the article entitled “A genome-scale CRISPR–Cas9 screening method for protein stability reveals novel regulators of Cdc25A,” recently published in *Cell Discovery*, our team developed a protein stability regulators screening assay (Pro-SRSA) by combining the whole-genome clustered regularly interspaced short palindromic repeats Cas9 (CRISPR–Cas9) library with a dual-fluorescence-based protein stability reporter and high-throughput sequencing to screen for regulators of protein stability. Based on our findings, we are confident that this efficient and unbiased screening method at the genome scale will be used by researchers worldwide to identify regulators of protein stability.

## Main text

Fine-tuned degradation of proteins plays a crucial role in a variety of physiological conditions, including cell cycle progression, cell growth, differentiation, and signaling transduction. The inappropriate degradation of some cancer-related proteins may induce cellular transformation and tumorigenesis [1]. Thus, understanding the precise regulation of protein degradation, especially for oncogenes and tumor suppressors, is important not only for understanding the biological mechanisms of cancer but also for developing therapeutic interventions. However, due to the lack of an efficient method, an unbiased and universal delineation of the regulators of protein homeostasis has not been previously possible. But now, based on the study reported in the article entitled “A genome-scale CRISPR–Cas9 screening method for protein stability reveals novel regulators of Cdc25A,” which was published in *Cell Discovery,* such an efficient method has been developed [2]. In this study, our team devised a protein stability regulators screening assay (Pro-SRSA) to screen for regulators of protein stability by combining the whole-genome clustered regularly interspaced short palindromic repeats Cas9 (CRISPR–Cas9) library with a dual-fluorescence-based protein stability reporter and high-throughput sequencing.

**Fig. 1:**
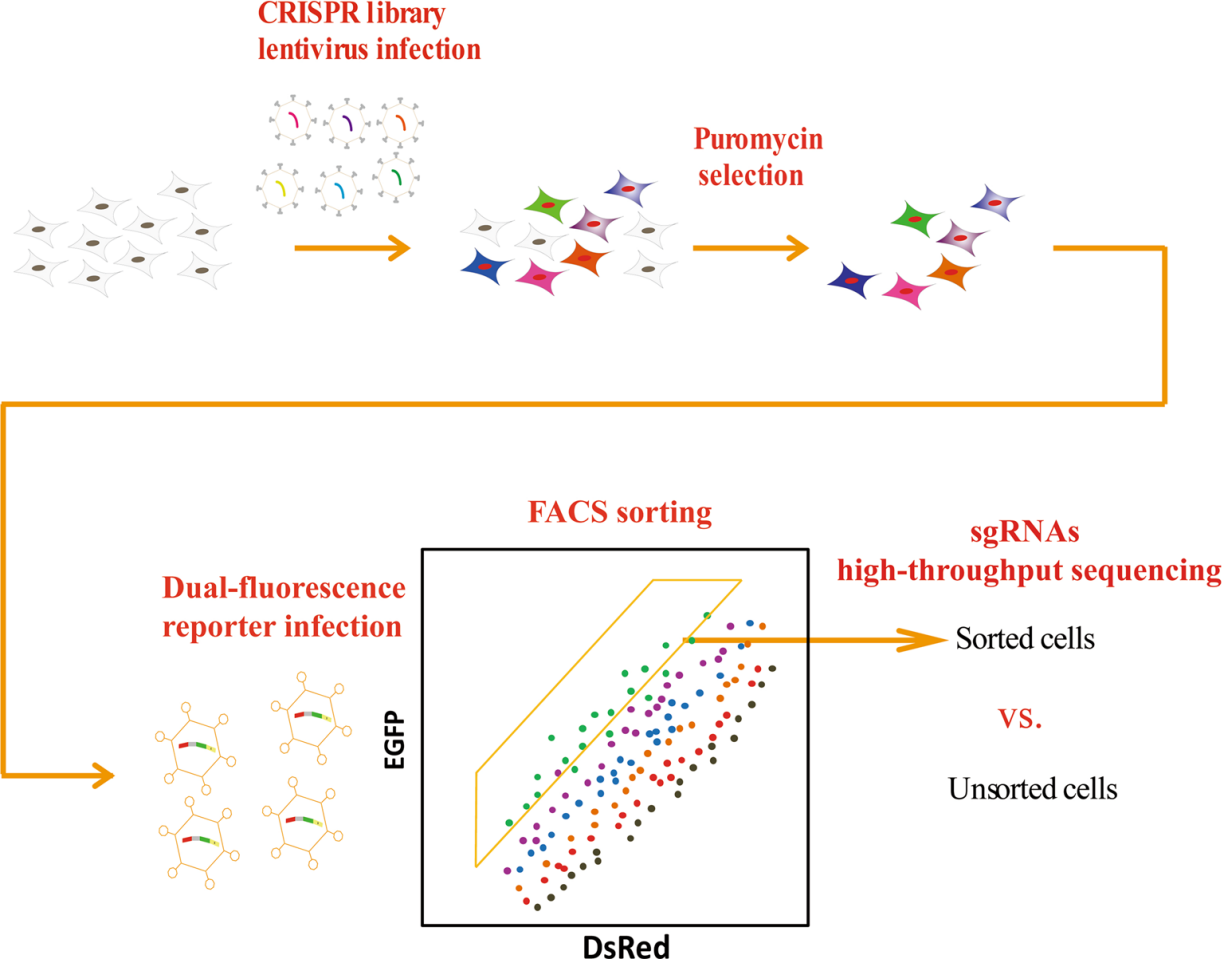
The workflow of the protein stability regulators screening assay (Pro-SRSA). *CRISPR* clustered regularly interspaced short palindromic repeat, *FACS* fluorescence-activated cell sorting, *EGFP* enhanced green fluorescence protein, *DsRed* *Discosoma* sp. red fluorescent protein, *sgRNA* single-guide RNA

The workflow of Pro-SRSA is divided into three steps (Fig. 1). The first step: genome-scale gene knockout. We infected the cells with lenti-CRISPR–Cas9 library at a low multiplicity of infection to control only one transgene copy number in most of the infected cells. Then, the cells were selected by puromycin for 1 week; during this period, the single-guide RNAs (sgRNAs) guided the Cas9 nuclease to cut the target sites, which introduced the indels and frame-shift of target genes through non-homologous end repair [3]. As a system for performing genome-scale loss-of-function screening, the CRISPR–Cas9 library was shown to be an easy and efficient method for gene knockouts [4, 5]. The second step: infection of a protein stability reporter. We developed an adenovirus reporter vector called pAd-DsRed-IRES-EGFP-X. In this dual-fluorescence reporter, a cassette containing DsRed-IRES-EGFP-X was used to reflect the stability of protein X. The internal ribosome entry site (IRES) permits translation of both *Discosoma* sp. red fluorescent protein (DsRed) and enhanced green fluorescence protein (EGFP)-X fusion proteins at a certain ratio [6]; knockout of any genes that affect the X protein stability would change the ratio of EGFP/DsRed, which would be monitored by flow cytometry. By combining the CRISPR–Cas9 library and the dual-fluorescence reporter, we were able to sort the cells with top ratio of EGFP/DsRed, then extract the genome DNA of the sorted and unsorted control cells and, by polymerase chain reaction, amplify the sgRNAs. Finally, the last step: high-throughput sequencing. By analyzing the enrichment of sgRNAs, we were able to determine which knockout contributed to the stabilization of X protein.

Using Pro-SRSA screening for cell division cycle 25A (Cdc25A) regulators, we found that cullin 4B (Cul4B)–damage-specific DNA binding protein 1 (DDB1)—DDB1 and CUL4 associated factor 8 (DCAF8) complex (Cul4B-DDB1^DCAF8^) is an E3 ligase for the degradation of Cdc25A. More interestingly, the screening also revealed that acetylation of Cdc25A at lysine 150, which is acetylated by E1A binding protein p300/CREB binding protein (p300/CBP) and deacetylated by histone deacetylase 3 (HDAC3), stabilizes the protein. In addition, using p53 as another example in our Pro-SRSA, we discovered a new degradation mechanism for p53 (manuscript in preparation).

## Conclusions

Pro-SRSA is a powerful screening method that could help researchers better understand cellular protein regulation in a variety of physiological and/or pathological processes, especially for the abnormal expression of oncogenes and tumor suppressors during tumorigenesis. Genome-scale screening for regulators of protein stability may open many new avenues for the treatment, perhaps even the curing, of diseases. Thus, we are confident that this easy and efficient method would be helpful for researchers focusing on protein degradation.
